# Nutritional substrates and microglial metabolic fitness in brain aging and Alzheimer's disease: from lipid handling to TREM2-linked translation

**DOI:** 10.3389/fnut.2026.1886340

**Published:** 2026-07-08

**Authors:** Ze Li, QiuLi Ming, LinPeng Fu, JingShuang Yang, YiLin Lv, BingQian Chen, ZhaoFeng Lu

**Affiliations:** 1 The First Affiliated Hospital and College of Clinical Medicine, Henan University of Science and Technology, Luoyang, China; 2 Third Affiliated Hospital of Henan Medical University, Xinxiang, China

**Keywords:** Alzheimer's disease, brain aging, immunometabolism, ketone bodies, lipids, microglia, TREM2

## Abstract

Alzheimer's disease is increasingly viewed as a disorder in which age-related disturbances in microglial metabolism and the handling of nutritional substrates contribute to progressive loss of protective function. This review examines how lipids and ketone bodies shape microglial metabolic fitness in the aging brain and in Alzheimer's disease, and how these effects intersect with triggering receptor expressed on myeloid cells 2 (TREM2) signaling and translational biomarkers. Available evidence indicates that early compensatory glycolysis may give way to chronic bioenergetic failure, while cholesterol and lipoprotein trafficking, lipid droplet accumulation, ketone-body signaling, and TREM2-associated lysosomal pathways influence plaque engagement, phagocytosis, and inflammatory responses. The review also considers how apolipoprotein E genotype, brain region, sex, disease stage, and model system condition translation from experimental models to human disease. Fluid, imaging, and tissue readouts are therefore discussed as stage- and context-dependent proxies rather than fixed signatures. Overall, nutritional strategies and microglia-targeted interventions are most likely to be informative when aligned with disease stage and biological context.

## 1 Introduction

### 1.1 Why microglial metabolic fitness matters in brain aging and Alzheimer's disease

Across single-nucleus datasets, brain aging leaves highly cell-type-specific marks, and microglia shows a distinct aging-related transcriptomic program (1). In human Alzheimer's disease, microglial populations are redistributed across disease stages and state programs; the central question is when those responses cease to match the demands of the tissue as pathology advances within the same lineage (2). Aged brains and apolipoprotein E (APOE4) Alzheimer's disease also accumulate an exhausted-like microglial population, tying age- and genotype-linked vulnerability to maladaptive state programs (3). Amyloid stress brings metabolism into focus, with an early glycolytic response in microglia giving way under chronic exposure to broader bioenergetic failure and impaired immune performance (4). TREM2 dosage sharpens the same point, tracking with state transitions, glucose uptake, phagocytic competence, and overall metabolic capacity across a graded response range (5). Important limits remain. Senescent TREM2-expressing microglia are present in aging and Alzheimer model mice, so stronger TREM2-linked programs cannot be treated as uniformly protective across genetic backgrounds, local niches, or disease windows in preclinical systems (6). Together, these findings justify asking whether substrate availability and handling, rather than inflammatory activation alone, help determine when Alzheimer's disease microglia retain protective function or progress toward metabolic failure (2, 4, 5).

### 1.2 Why nutritional substrates are central to microglial metabolic fitness

In this review, nutritional substrates are used in a deliberately broad but defined sense. They include diet-derived lipid and ketone substrates, diet-sensitive circulating metabolites and lipoproteins, and brain-resident substrate pools that are remodeled by aging, APOE genotype, amyloid burden, and cellular stress. This distinction is important because endogenous lipid remodeling in Alzheimer's disease microglia is not equivalent to direct dietary lipid delivery, and human dietary interventions usually modify the systemic metabolic milieu before any brain-resident microglial effect can be inferred (7).

Systemic nutritional status also provides a clinically relevant entry point. In a mouse model fed a high-fat fructose diet, liver injury was accompanied by altered serum lipid and glutamate levels. The same model also showed increased inflammatory cytokines in liver, hippocampus, and cortex, together with cortical and hippocampal astrocyte and microglial activation. These findings support a liver–brain-axis view in which diet-induced peripheral metabolic stress can lower central resilience and prime glial responses (8). A broader review of microglial metabolism places nutritional interventions, ketone bodies, pharmacological metabolic modulators, and gut-microbiota approaches within a shared framework of neuroinflammatory regulation (9). A pharmacological neuroprotection study using sigma-1 receptor modulation by clemastine further illustrates that stress-response, inflammatory, and oxidative pathways can be therapeutically modified, although such evidence should be treated as complementary rather than nutrition-specific (10).

Nutritional substrates offer a useful mechanistic entry point; damaging lipid droplets in APOE4/4 human Alzheimer's disease microglia tie lipid burden to maladaptive state programs and show that nutrient availability, storage, and handling actively shape microglial responses (11). Triglyceride metabolism is likewise required for inflammatory and microglial phenotypes associated with APOE4, placing substrate handling directly on the axis of state formation (12). Postmortem metabolomics points in the same direction, with Alzheimer brain tissue showing broad deficits in energy-metabolite availability, although this evidence alone cannot localize the disturbance to microglia or establish a cell-specific mechanism (13). β-hydroxybutyrate (BHB) extends the argument: in human microglia challenged with amyloid-β (Aβ) oligomers, this ketone body reshapes metabolism and alters programs that support disease-relevant behavior (14). Human dietary intervention studies are consistent with that view. A modified Mediterranean ketogenic diet reverses an Alzheimer-like peripheral lipid signature even though direct validation in brain-resident microglia remains unavailable (7). Preclinical work in APP/PS1 mice reaches a similar endpoint, with long-term ketogenic diet and exogenous BHB rescuing hippocampal long-term potentiation while direct microglial validation remains limited (15).

### 1.3 Search strategy and reading the evidence

This is a narrative review with a structured literature-identification strategy. We searched PubMed, Web of Science, and Google Scholar for English-language articles using combinations of the terms “Alzheimer's disease,” “brain aging,” “microglia,” “microglial metabolism,” “immunometabolism,” “lipid droplets,” “APOE,” “TREM2,” “ketone bodies,” “β-hydroxybutyrate,” “medium-chain triglycerides,” “ketogenic diet,” “biomarkers,” and “nutrition intervention.” We used a recent-weighted rather than date-restricted approach: literature published from 2022 to 2026 was prioritized to capture current advances, while older mechanistic, methodological, or landmark studies were retained when they defined key pathways, assays, concepts, or intervention frameworks needed to interpret the newer evidence. We prioritized human tissue, human biomarker, and human intervention studies where available, and used animal and cellular studies to clarify mechanisms that remain difficult to test directly in human brain-resident microglia.

The Alzheimer's literature now spans many analyte classes, disease stages, cohorts, and assay settings, so a broad inflammatory inventory often adds breadth without adding much clarity (16). Signals that remain reproducible across cohorts and clinical stages deserve special weight, in line with current performance-based recommendations for biomarker use in specialized care (17). State labels also need to be used operationally. Long-read RNA sequencing in human microglia reveals extensive disease-associated isoform regulation and shows that labels shift with analytic resolution and dataset structure (18). The same discipline applies to interpretation, and even strong human associations do not establish causality or deliver a one-to-one map of brain-resident microglial states (19). Cross-dementia datasets still sharpen the language of state change; shared MSR1-linked phagocytic programs can supply a transferable vocabulary without being mistaken for Alzheimer-specific pathology (20). Preclinical mechanistic studies, human biomarker readouts, and clinical-trial evidence answer different translational questions and should not be treated as interchangeable evidentiary levels (21).

Nutritional substrate availability and handling are treated here as upstream constraints on microglial metabolic fitness. Microglial metabolic fitness, in turn, shapes how cells engage plaques, lipid stress, inflammatory tone, and TREM2-linked programs, whereas human fluid, imaging, and tissue measures provide imperfect but useful proxies for these substrate–state relationships. We interpret the evidence hierarchically: human dietary intervention studies are most informative for clinical feasibility and host-level metabolic response; human fluid, imaging, and tissue studies provide translational anchors but often remain associative or compartment-limited; animal models test causal mechanisms and disease windows; and *in vitro* microglial systems provide mechanistic resolution but should not be placed on the same evidentiary level as human intervention or tissue data. Thus, dietary and peripheral metabolic findings can suggest plausible links to microglial biology, but they do not by themselves establish brain-resident microglial remodeling without support from human tissue, imaging, or cell-specific data.

## 2 Defining microglial metabolic fitness

### 2.1 What microglial metabolic fitness refers to

In this review, microglial metabolic fitness is used as an operational term rather than as a single marker, cluster label, or activation state. It refers to the capacity of microglia, within a defined brain region and disease stage, to maintain energy production, substrate handling, organelle quality control, and functional output under aging- and Alzheimer's disease-related stress. We evaluate this capacity across four domains: metabolic programs; lysosomal, mitochondrial, and lipid-cargo processing; effector functions such as phagocytosis, plaque engagement, and inflammatory restraint; and translational interpretability through fluid, imaging, and tissue proxies. This framing allows later sections to connect state descriptors with metabolic programs and functional consequences.

Microglial metabolic fitness cannot be reduced to activation status alone. In plaque-associated microglia, protective counterprograms remain visible, and disrupting them intensifies inflammatory tone and amyloid burden (22). It is not a fixed cluster label, either. Living human microglia sampled across central nervous system (CNS) regions and disease contexts occupy disease-enriched subsets that shift with tissue environment and experimental perturbation (23). Amyloid-linked settings make the same point. Xenografted human microglia adopts multiple transcriptomic states in response to Alzheimer-related Aβ pathology (24). Any workable definition therefore has to lean on function, not marker lists alone. Disruption of MS4A6A/Ms4a6d alters plaque engagement, plaque wrapping, phagocytic competence, and inflammatory balance *in vivo* in Alzheimer models (25). It also requires explicit coupling between metabolic state and functional output. In Alzheimer's disease, glycolytic reprogramming can coexist with phagocytic exhaustion and a proinflammatory state under stress (26). The term becomes most useful when state descriptors, dominant metabolic programs, and major functions are read together, with human relevance still in view (27).

### 2.2 Homeostasis, aging, and disease-associated continua

Homeostatic microglia is not a passive default. In Alzheimer-related settings, T cell immunoglobulin and mucin domain-containing protein 3 (TIM-3)-dependent checkpoint signaling helps preserve core microglial identity and restrain inflammatory deviation (28). Human single-nucleus studies show that, as pathology advances, microglia are redistributed across multiple state programs, making departure from homeostatic identity look like staged reweighting along a continuum instead of abrupt replacement by one activated phenotype (2). No single axis organizes that continuum. In human and mouse single-nucleus datasets, TREM2-dependent and TREM2-independent responses separate from one another, indicating partially divergent routes into disease-associated programs (29). Aging-related branches intersect with these continua without collapsing into them; Alzheimer-linked microglial states remain vulnerable to senescence and cholesterol dysregulation, which can amplify and reshape the repertoire of disease-associated responses (30). Spatial and chromatin-resolved profiling in amyloid model mice extends the same view, with non-plaque-associated and plaque-associated states showing dynamic, regionally structured relationships instead of converging on a single terminal disease-associated microglial endpoint (31). Stress-linked branches also belong here. Dark microglia coupled to toxic lipid secretion mark a neurodegenerative response program with distinct structural and metabolic features (32). Taken together, these studies support a continuum model in which aging, plaque proximity, lipid stress, and senescence reweight microglial states rather than creating a single terminal Alzheimer's disease phenotype.

### 2.3 Glycolysis, OXPHOS, lipid handling, and lysosome–mitochondria coupling

In Alzheimer-related microglial stress, glycolysis behaves as an inducible core module that eventually fails under sustained pressure, with early amyloid-driven compensation giving way to broad bioenergetic collapse and weaker phagocytic and immune performance (4). Disturbed glucose handling sits on the same axis of state transition. In experimental systems, glucose dyshomeostasis pushes microglia away from homeostatic programs toward an early disease-associated stage (33). Even that axis is not linear. Manipulating hexokinase 2 shows that partial and near-complete suppression do not produce the same consequences for microglial activation or Alzheimer progression (34). Oxidative phosphorylation (OXPHOS) becomes more informative when anchored to respiratory capacity. The protective PLCγ2-P522R variant in human induced pluripotent stem cell (iPSC)-derived microglia is linked to higher respiration alongside a more favorable functional profile (35). Lipid handling involves more than the presence of droplets, with arachidonic acid mobilization and peroxidation directly promoting microglial dysfunction in Aβ pathology (36). Lysosome–mitochondria coupling also shifts under stress. In human iPSC-derived microglia, inflammation-driven reprogramming imposes lysosomal dysfunction, and the defect deepens in the APOE4/4 genotype (37). Specific endolysosomal trafficking machinery matters as well: loss of the Alzheimer-linked gene SORL1 impairs lysosomal degradation, lysosomal enzyme activity, and exocytic handling in human microglia (38). These modules are analytically separable, but within cells they remain tightly coupled and intersect across carbon metabolism, organelle stress, and effector function (39).

### 2.4 Brain region, sex, genotype, model system, and disease stage

**Figure 1:**
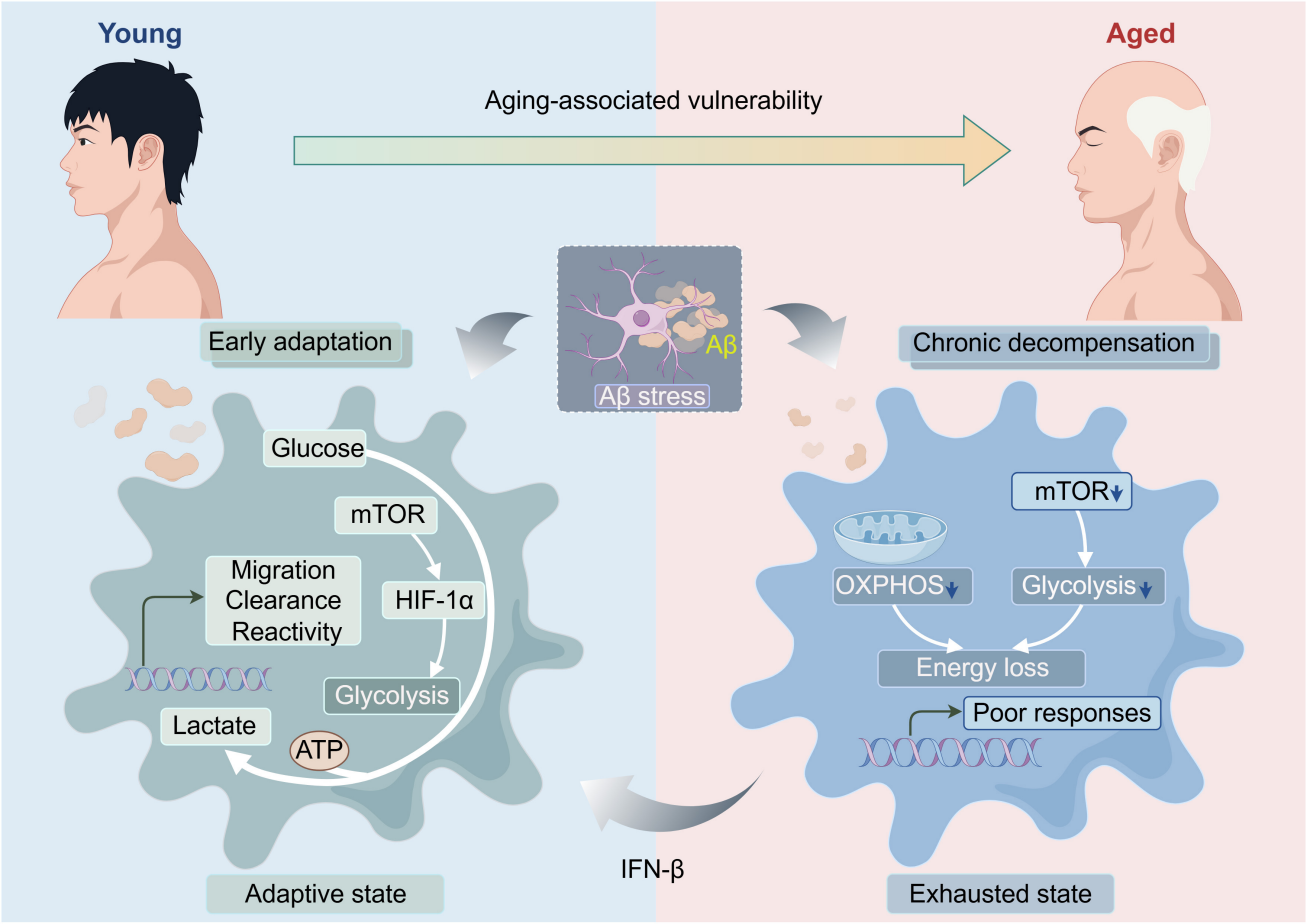
Aging shapes microglial metabolic fitness across the Alzheimer's disease continuum. This schematic is a conceptual, non-linear model of how aging and persistent Aβ stress may reshape microglial metabolic fitness across disease-relevant contexts; it is not intended to imply a single uniform trajectory from young to aged or from adaptive to exhausted states. In the early phase, microglia may show glycolytic compensation associated with glucose use, lactate production, ATP maintenance, and adaptive migration, clearance, and reactivity. With sustained stress, mTOR signaling, oxidative phosphorylation, and glycolytic compensation may decline, contributing to energy loss, poor responses, and exhausted-like features. The balance among these states is expected to vary with brain region, sex, genotype, pathology burden, disease stage, and model system, as discussed in Section 2. The figure was created using Figdraw. Aβ, amyloid-β; AD, Alzheimer's disease; HIF-1α, hypoxia-inducible factor 1 alpha; IFN-β, interferon beta; mTOR, mechanistic target of rapamycin; OXPHOS, oxidative phosphorylation; ATP, adenosine triphosphate. Created by Figdraw.com.

Judging microglial fitness starts with disease stage. Homeostatic microglia participates in early plaque seeding, whereas later activated microglia contributes to plaque reshaping and compaction (40). The same lineage can therefore look maladaptive or plaque-restraining depending on when it is sampled. Brain region then adds a second layer: single-nucleus and spatial profiling of human temporal cortex and white matter reveal distinct state–pathology associations across gray- and white-matter compartments (41). Even within a given region, micro-anatomical context matters. In the middle temporal gyrus, layer-specific microglia–plaque colocalization is linked to cognitive decline (42). Local niche conditions further reshape the picture. Plaque-proximal hypoxia drives hypoxia-inducible factor 1 alpha (HIF-1α)-linked mitochondrial dysfunction in Alzheimer's disease microglia (43). Sex is not a background covariate to average away. Female 5xFAD mice show stronger microglial interferon signaling together with greater Aβ plaque pathology, independent of estrous cycle stage (44). Circulating hormones do not fully account for that effect. Sex chromosomes and gonads independently influence microgliosis, plaque burden, plaque remodeling, and CD11c-positive microglial responses (45). Genotype adds yet another layer: Apolipoprotein E (ApoE) isoforms differentially reshape the transcriptomic and epigenomic landscapes of human microglia in amyloid-bearing xenograft systems and bias migration, phagocytic, and immune-response programs in distinct directions (46). Model system must stay in view as well. Transplantation platforms and host App backgrounds delimit the response space available to human microglia, making fitness judgments non-transferable across models even when similar state labels are used (47) (Figure 1 and Table 1).

**Table 1:** Microglial metabolic fitness across brain aging and Alzheimer's disease.

Context/state	Metabolic profile	Functional role	Human support	Key limitation	References
Homeostatic-to-AD continuum	Stage-linked shift from surveillance to inflammatory and lipid-responsive programs	Defines state transition landscape	Direct human single-nucleus RNA sequencing/spatial support	Labels remain context-dependent	(2)
APOE4 lipid-burdened state	Lipid droplet accumulation and impaired lipid handling	Links APOE4 to damaging microglial lipid stress	Human AD APOE4/4 microglia	Mainly association/state mapping	(11)
Acute glycolytic adaptation → chronic collapse	Early glycolytic shift; later energetic failure	Separates adaptation from decompensation	Human overlap indirect	Causal basis mainly preclinical	(4)
Lipid-droplet-accumulating aging state	Lipid droplets, ROS burden, impaired catabolism	Links aging lipid stress to dysfunction	Aging human-linked support	Not a standalone state definition	(66)
TREM2-dependent/independent AD response	Divergent activation programs across AD	Defines TREM2-linked state branching	Human and mouse single-nucleus data	Does not resolve all state overlap	(29)
TREM2–lysosomal competence state	Impaired lysosome and lipid trafficking with TREM2 loss	Links TREM2 to metabolic competence	Human iPSC-derived microglia/variant relevance	Model still reductionist	(75)
Stage-dependent plaque response	Early seeding; later compaction	Explains opposite plaque effects across stages	Humanized/pathology-linked support	Strong stage dependence	(40)
APOE4 lipid-overload surveillance failure	Lipid accumulation with impaired surveillance	Links APOE4 to network-monitoring defects	Human iPSC-derived microglia	Cellular model only	(57)
Microglia-dependent lipid remodeling	Microglia-linked vs. non-microglial lipid changes	Refines lipid-source attribution	Preclinical causal support	Human confirmation limited	(52)
Hypoxia-stressed plaque-response state	Mitochondrial stress under HIF1-linked hypoxia	Links oxygen stress to reduced fitness	AD-relevant preclinical model	Human evidence indirect	(43)
Protective counterstate	Lymphoid-like protective program	Counters one-axis “activation = harm” model	Human-linked support	Mechanistic boundary still emerging	(22)
Region- and niche-dependent state variation	Cortex–white matter spatial heterogeneity	Adds brain-region context to state calls	Direct human spatial support	Spatial association, not intervention	(41)
Tau-linked lipid-droplet maladaptation	Inflammatory and lipid-droplet program with impaired tau handling	Connects metabolic maladaptation to tauopathy	Mainly preclinical	Sex-dependent and model-dependent	(74)
ApoE/lipoprotein sensing state	Isoform-specific phospholipid and lipoprotein handling	Shapes migration, uptake, and Aβ response	Mechanistic preclinical support	Human translation indirect	(51)

This table provides a working framework for microglial metabolic fitness across brain aging and Alzheimer's disease. The listed states are not mutually exclusive or exhaustive; rather, they summarize recurrent state–metabolism–function configurations discussed in the main text and should be interpreted in relation to disease stage, anatomical niche, genotype, and model system. “Human support” indicates the degree of direct human anchoring, whereas “Key limitation” highlights the main evidentiary or translational boundary for each row. References are representative rather than exhaustive, with one key study listed per row. Representative intervention relevance is summarized in Table 3. AD, Alzheimer's disease; Aβ, amyloid-β; APOE, apolipoprotein E genotype; ApoE, apolipoprotein E protein; iPSC, induced pluripotent stem cell; OXPHOS, oxidative phosphorylation; ROS, reactive oxygen species; TREM2, triggering receptor expressed on myeloid cells 2.

## 3 Lipid availability, handling, and maladaptation

### 3.1 Uptake, trafficking, storage, and oxidation

Dietary fatty acids enter this pathway by shaping microglial polarization in Alzheimer's disease-related settings (48). Fatty-acid class matters: palmitate favors inflammatory responses, whereas docosahexaenoic acid (DHA) can blunt several of these changes in microglial models (49). Within the n-3 axis, specialized pro-resolving mediators derived from dietary polyunsaturated fatty acids may support microglial mitochondrial respiration and inflammatory resolution in Alzheimer's disease models (50).

Lipid dysregulation begins before overt droplet accumulation. In preclinical Alzheimer models, extracellular ApoE-containing lipoprotein phospholipids engage microglia in an isoform-specific manner and shape both migration and Aβ uptake (51). Trafficking shows the same selectivity. Microglial depletion in 5xFAD mice alters only a subset of disease-linked lipid species, indicating that microglia help move, remodel, and redistribute particular lipid pools (52). Storage marks an active metabolic turn, with Aβ exposure driving a DGAT2-dependent shift from free fatty acids toward triglyceride synthesis and lipid droplet formation that coincides with phagocytic dysfunction (53). Genotype further conditions that state. APOE4 reshapes the lipid-droplet proteome and alters inflammatory output in microglia, so droplet accumulation does not carry one fixed functional meaning (54). Lipid burden also feeds back on effector competence. Reducing microglial lipid load in an Alzheimer mouse model enhances phagocytosis and improves amyloid handling (55). Oxidized cholesterol metabolites extend the picture beyond neutral storage: 25-hydroxycholesterol impairs microglial surveillance and phagocytosis while worsening Alzheimer-related pathology through cholesterol esterification (56). Together, these findings place dietary and endogenous lipid signals on a shared lipid-handling axis, while leaving direct links between specific human dietary lipid exposures and brain-resident microglial remodeling largely unresolved.

### 3.2 Cholesterol, phospholipids, ApoE, and lipoprotein pathways

Cholesterol and phospholipid traffic provide the main thread through the ApoE/lipoprotein pathway. In human Alzheimer's disease, APOE4/4 is linked to a damaging lipid-droplet microglial state, placing cholesterol and phospholipid handling inside a genotype-conditioned pathway stress response (11). Experimental work reaches the same functional endpoint. APOE4-driven lipid accumulation impairs microglial surveillance of neuronal-network activity (57). The ApoE/lipoprotein pathway also sits inside the pathogenic circuit, with microglial ApoE particles contributing to neuronal senescence and synaptotoxicity (58). That sequence extends to ApoE aggregation itself. In experimental systems, microglial uptake and aggregation of ApoE can seed β-amyloidosis (59). Excess cholesterol adds another layer of dysfunction, weakening microglial clustering and activation around amyloid plaques and changing how efficiently plaques are engaged (60). Downstream handling matters too. Excessive cerebral cholesterol ester accumulation in APPNL-G-F mice parallels reduced microglial recruitment and phagocytosis (61). Genotype alone still does not define the ApoE/lipoprotein pathway. ApoE biology varies with secreting cell type as well as isoform, and that distinction matters when cholesterol and phospholipid trafficking are interpreted in Alzheimer's disease (62). A recent review now frames microglial lipid and lipoprotein metabolism not only as a pathology axis but also as a potential intervention axis in Alzheimer's disease (63). Lipidomics argues for the same caution. Some APOE4-linked cholesterol ester signatures may reflect astrocyte-dominant lipid states and should not be reassigned to microglial cholesterol handling when cellular source attribution remains unresolved (64). Circulating lipoproteins can carry lipid mediators with inflammatory activity, but current CNS data are still too limited to treat peripheral lipid signals as direct evidence of brain-resident microglial lipid remodeling (65).

### 3.3 Lipid droplets, lysosomal stress, and plaque-associated microglia

Local brain lipid stress becomes most visible at the level of lipid droplets and plaque-associated lysosomal cargo. Lipid droplets do more than store neutral lipid. In aging brain, they mark a microglial state that is functionally compromised and proinflammatory (66). That background becomes directly relevant in plaque environments, where human plaque-associated microglia accumulate lipid droplets in a chimeric Alzheimer model (67). In an App knock-in amyloid model, microglia containing intracellular fibrillar Aβ acquire a foam-cell-like state with broad metabolic dysregulation (68). Lysosomal stress enters through plaque-associated cargo handling. P2X4 and ApoE converge in plaque-associated microglia, and P2X4 promotes ApoE degradation through lysosomal cathepsin B (69). Efficient plaque handling also depends on endolysosomal trafficking, with VPS35-dependent TREM2 recycling supporting Aβ endocytosis by plaque-associated microglia in murine Alzheimer's disease (70). bis(monoacylglycerol)phosphate (BMP) and lysophospholipids are most informative here as plaque-microenvironment signals. Mass spectrometry imaging shows that these lipid classes accumulate around Aβ plaques and intensify with pathological progression (71). Even within plaque-bearing tissue, lipid chemistry remains heterogeneous. Single-plaque multimodal chemical imaging reveals distinct lipid–amyloid configurations across individual plaques in transgenic models (72). Network-level analyses support the same view, placing BMP, LPC, and LPE within a lysosomal stress architecture after amyloid exposure in 5xFAD cortex and hippocampus (73). Nor is droplet stress confined to amyloid systems; microglial REV-ERBα drives lipid droplet formation together with inflammatory pathology in male tauopathy mice (74). Thus, plaque-associated lipid droplets and lysosomal lipid species are best interpreted as local stress readouts rather than direct proxies for dietary lipid intake.

### 3.4 Lipid stress and TREM2-linked programs

**Figure 2:**
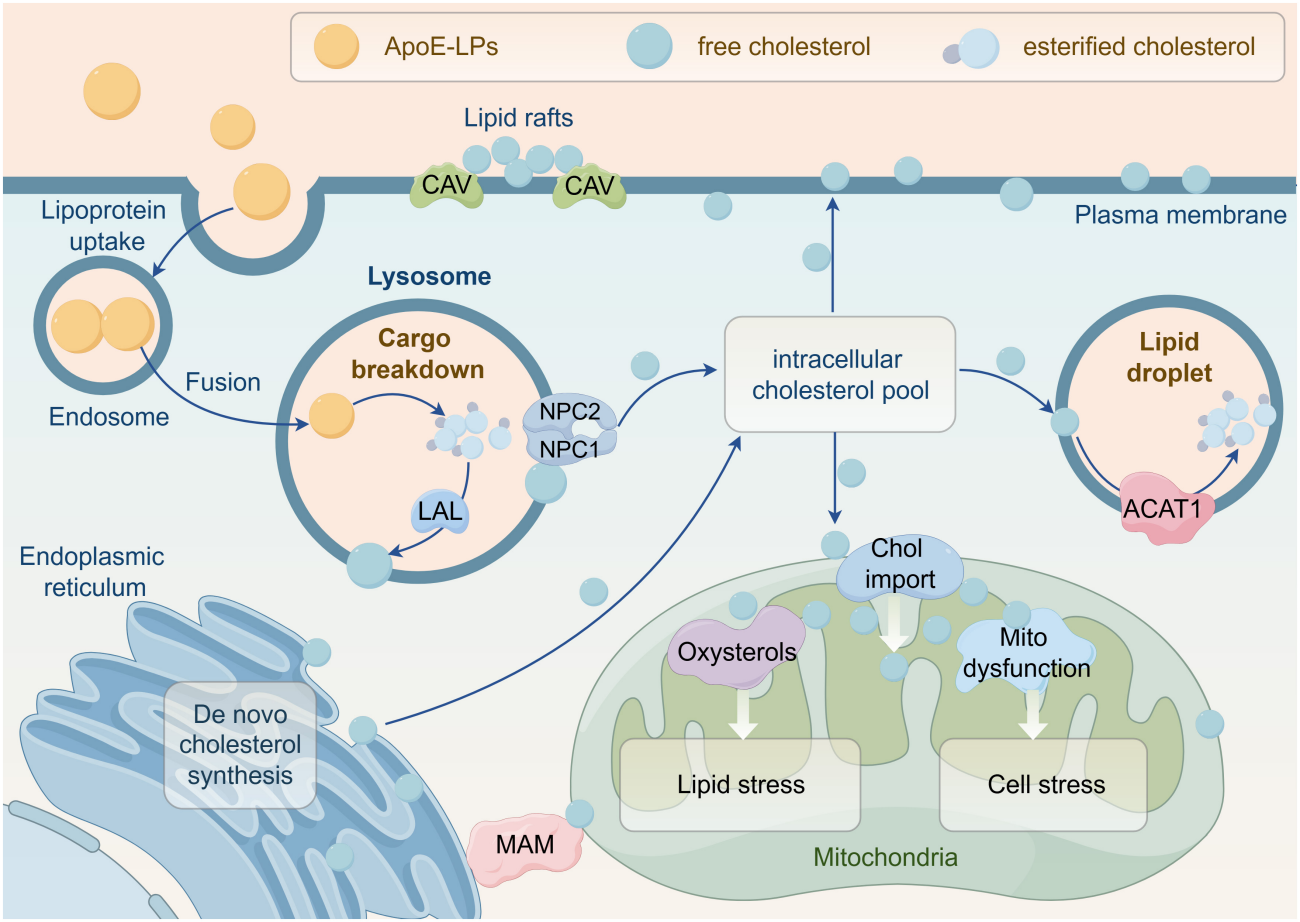
Cholesterol-centered lipid handling and lipid-droplet stress in microglia. This schematic summarizes a cholesterol-centered framework for microglial lipid uptake, trafficking, storage, and maladaptation. ApoE-containing lipoproteins are taken up into endolysosomal compartments, where cargo breakdown and lysosomal acid lipase release cholesterol that is exported by NPC1/2 to an intracellular cholesterol pool. Cholesterol is then redistributed to the plasma membrane, stored as cholesteryl esters in lipid droplets via ACAT1, and supplemented by *de novo* synthesis in the endoplasmic reticulum. Excess cholesterol burden is linked to oxysterol accumulation, mitochondrial dysfunction, and broader cellular stress. Processes involving lipoprotein uptake, lysosomal cholesterol processing, NPC1/2-dependent export, ACAT1-linked esterification, and lipid-droplet accumulation are supported most directly in microglia, whereas membrane-domain organization, some aspects of intracellular redistribution, and the downstream stress-routing modules are shown schematically as mechanism-informed inferences from broader lipid biology and related neurodegenerative literature. The figure was created using Figdraw. ApoE-LPs, apolipoprotein E-containing lipoproteins; ACAT1, acyl-CoA:cholesterol acyltransferase 1; CAV, caveolae/caveolin-rich membrane domains; Chol, cholesterol; LAL, lysosomal acid lipase; MAM, mitochondria-associated membrane; NPC1/2, Niemann–Pick disease type C1/C2 proteins. Created by Figdraw.com.

Within TREM2-linked lipid stress, extracellular lipoprotein burden and intracellular cholesterol-handling failure converge at the lysosome–cargo interface. Human microglia carrying a TREM2 loss-of-function mutation show lysosomal failure, abnormal cholesterol handling, and broader defects in lipid homeostasis (75). ApoE provides a direct receptor-level route into the same stress program. As a TREM2 ligand, it allows extracellular lipoprotein burden to be sensed at the cell surface (76). Amyloid stress meets that pathway at the same interface. ApoE4 and oligomeric Aβ42 engage overlapping binding surfaces on TREM2, so lipoprotein and amyloid stress can converge before downstream programs diverge (77). Ligand richness still does not guarantee effective signaling, however. Alzheimer-associated variants impair TREM2 multimerization and weaken receptor output even in ligand-rich environments (78). Downstream signaling also branches, with microglial responses to Aβ requiring both SYK-dependent and SYK-independent TREM2 pathways (79). Cholesterol handling feeds back on the same architecture. ACAT1 inhibition increases soluble TREM2 (sTREM2) shedding while enhancing LRP1-dependent Aβ phagocytosis in microglia (80). Stronger TREM2 activity is therefore not automatically beneficial; a gain-of-function TREM2 variant can still produce maladaptive microglial behavior (81). A broader lipid-recycling context supports the same conclusion, placing TREM2 within a wider axis that links cholesterol efflux, lipid-droplet homeostasis, and microglial adaptation across neurodegenerative settings (39) (Figure 2).

### 3.5 Dietary lipids, peripheral lipoproteins, and brain-resident lipid stress

One useful boundary is between lipid exposure and brain-resident lipid stress. Dietary lipid exposure may shape the host lipid milieu, fatty-acid composition, and inflammatory tone, but consumed lipid should not be assumed to map directly onto plaque-associated microglial lipid stores. Fatty-acid class illustrates the point: palmitate and DHA drive different microglial responses in model systems, so dietary lipids should not be treated as a single biological category (49). The n-3/DHA axis adds a resolution-oriented layer, because n-3-derived specialized pro-resolving mediators have been linked to microglial mitochondrial respiration and inflammatory resolution in Alzheimer's disease models (50). By contrast, cholesterol and ApoE-containing lipoproteins primarily define a brain-resident handling axis involving transport, trafficking, lysosomal processing, and local cellular stress, even as microglial lipid and lipoprotein metabolism is increasingly discussed as an intervention axis (63). Peripheral lipoproteins may carry inflammatory lipid mediators, but current CNS evidence does not show that peripheral lipid changes directly remodel brain-resident microglial lipid droplets, plaque-associated lysosomal lipid stress, or TREM2-linked cargo handling (65). Dietary lipid interventions are therefore best framed as modifiers of the systemic lipid environment and inflammatory tone; direct effects on human microglial lipid handling remain questions for biomarker-linked and tissue-resolved studies.

## 4 Ketone bodies in brain aging and Alzheimer's disease

### 4.1 Ketone availability in aging and Alzheimer's disease

In Alzheimer's disease, ketone availability appears reduced but not abolished. Ketogenic medium-chain triglycerides increase brain ketone uptake in patients with mild-to-moderate disease despite persistent global energetic vulnerability (82). Clinical evidence from mild cognitive impairment (MCI) points in the same direction, with a 6-month ketogenic drink improving cognition even though it does not identify the relevant cell-specific cerebral mechanism (83). Aging does not seem to eliminate responsiveness to ketone-oriented substrate manipulation; medium-chain triglycerides alter gait performance together with brain metabolic network measures in healthy older adults, although those readouts still fall short of direct quantification of cerebral ketone uptake (84). Experimental work likewise favors a shortfall model over complete loss. Restoration of the HMGCS2-dependent BHB–H3K9bhb axis ameliorates synaptic plasticity and cognition in an Alzheimer model (85). Availability, however, should remain distinct from downstream mechanism. Medium-chain triglycerides can improve cognition and systemic metabolism in Alzheimer mouse models without proportional increases in circulating ketones (86).

### 4.2 Ketone signaling in microglia

In microglia, ketone bodies act as signaling metabolites, with BHB directly reprogramming human microglial metabolism under Aβ oligomer challenge while suppressing inflammatory output (14). Among downstream pathways, the NLRP3 inflammasome remains the clearest candidate node linking ketone signaling to inflammatory restraint in Alzheimer-related systems (87). Mitochondrial efficiency also needs a precise meaning. In activated microglia, BHB oxidation drives accumulation of immunometabolites, indicating that ketolysis reroutes carbon flux in ways that can reshape immune output, biosynthetic balance, and stress adaptation (88). That rerouting becomes especially relevant under substrate stress. A ketone diester preserves acetyl-CoA, ATP, mitochondrial membrane potential, and ketolytic enzyme activity in microglial cells exposed to low glucose and Aβ (89). Redox control maps onto competence as well, with BHB improving redox status together with cytokine handling and phagocytic potency in glucose-deprived human microglia-like cells (90). Boundaries remain essential. The anti-inflammatory effect of BHB is concentration-dependent and reversible with blockade of monocarboxylate transport, and most current support still comes from cultured microglial systems (91).

### 4.3 Limits of the current evidence

Human intervention studies already show that ketone delivery can modify brain energetics in Alzheimer's disease, yet the resulting inference stays narrow: ketogenic medium-chain triglycerides increase brain ketone uptake in mild-to-moderate disease, but the readouts remain whole-brain metabolic measures (82). Clinical benefit still falls short of mechanistic resolution. A 6-month ketogenic drink improves cognition in MCI without accompanying microglial or inflammatory biomarkers (83). The same gap persists when human studies add serum ketones, plasma Aβ42, or mitochondrial DNA markers. Supplementation with medium-chain triglycerides combined with DHA broadens the substrate context beyond ketone delivery alone (92). However, the resulting evidence remains peripheral and clinically indirect rather than direct proof of brain-resident microglial remodeling. Preclinical support also remains model-contingent. In female APOE4 mice, a ketogenic diet improves memory, indicating that sex and genotype can condition response magnitude and direction (93). Benefit may arise through routes that are not primarily microglial. Coconut oil-derived medium-chain triglycerides in 5xFAD mice are linked to neurite outgrowth and maintenance of gut homeostasis alongside memory improvement (94). Even when ketogenic or related dietary interventions converge on favorable phenotypes, they remain mechanistically heterogeneous and clinically variable (95). Broader inflammatory-mediator frameworks, including HMGB1–TLR4/RAGE signaling, also suggest that metabolic interventions may intersect with neuroinflammatory pathways beyond ketone delivery alone, although current support remains indirect and largely outside Alzheimer-specific intervention studies (96). This preclinical therapeutic landscape is best interpreted as complementary to—rather than mechanistically equivalent to—ketone- or MCT-based substrate interventions.

## 5 TREM2 as a mechanistic bridge between lipid stress and state transition

### 5.1 Signaling, dosage, and state transition

TREM2 signaling operates along a graded axis, with variation in receptor expression tracking microglial state, glucose uptake, phagocytic competence, and overall metabolic capacity across defined response windows (5). Receptor dosage alone does not determine that range. PLCG2 modulates both TREM2 expression and downstream signaling in the presence of Alzheimer-related pathology (97). Expression level is itself genetically regulated. MS4A4A and MS4A6A cooperate to negatively regulate both transmembrane and sTREM2 while reshaping microglial states (98). Biochemical state matters as well. NEU1-governed Trem2 sialylation determines full-length receptor processing and favors Trem2-DAP12-Syk signaling (99). Stage then reshapes the relationship between TREM2 dosage and state conversion. Elevating microglial TREM2 reduces amyloid seeding and suppresses disease-associated microglia at early seeding stages (100). Experimental agonism shows that receptor strength is actionable: a fully human high-affinity anti-TREM2 antibody enhances p-SYK activation, improves stressed human microglial viability, and lowers plaque burden in a clinically relevant Alzheimer model (101). Stronger TREM2 output still does not guarantee uniformly protective state conversion. In a tau model, TREM2 overexpression produces robust microglial activation but only modest mitigation of tau-linked injury (102).

### 5.2 Shedding, soluble TREM2, and therapeutic modulation

Therapeutic modulation enters the TREM2 axis as a measurable pharmacodynamic process. In first-in-human evaluation of AL002, cerebrospinal fluid (CSF) target-engagement changes included reduced sTREM2 together with shifts in myeloid-associated markers (103). Brain delivery then becomes part of the mechanism itself. A TREM2-activating antibody linked to a blood–brain barrier transport vehicle improves brain penetration, reshapes microglial state, and enhances metabolic responsiveness in Alzheimer's disease models (104). Shedding also belongs to a regulated processing pathway, with RHBDF2 modifying microglial TREM2 proteolysis (105). Beneficial modulation does not point to a single direction of sTREM2 change, however. A ligand-mimetic anti-TREM2 agonist antibody can preserve physiological shedding, elevate sTREM2, and still ameliorate pathology in preclinical models (106). sTREM2 becomes easier to interpret once it is treated as a potentially bioactive branch. Released ectodomain can ameliorate tau phosphorylation and cognitive deficits through transgelin-2 in an Alzheimer model (107). Human interpretation remains conditional. MS4A-cluster variants modify how sTREM2 levels relate to neuropathology biomarkers, implying that the same apparent sTREM2 level may not carry the same biological meaning across genetic backgrounds (108). Consistent with that complexity, higher CSF sTREM2 is associated with slower hippocampal atrophy and slower cognitive decline independently of p-tau181 (109). Quantity alone still falls short. TREM2 splice isoforms generate distinct soluble species that disrupt long-term potentiation (110).

### 5.3 Stage-, dose-, and context-dependent effects

**Figure 3:**
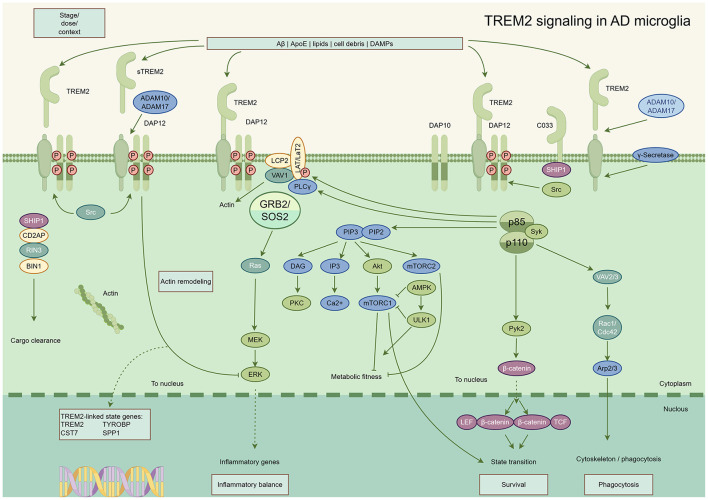
Selected TREM2 signaling modules linking lipid stress to microglial state transition, cargo handling, and metabolic fitness in Alzheimer's disease. This schematic is intended as a selected, non-exhaustive overview rather than a complete signaling map. Ligands associated with amyloid pathology and lipid stress, including Aβ, ApoE-containing lipoproteins, lipids, cell debris, and DAMPs, converge on TREM2 in AD microglia. TREM2 signals mainly through DAP12 and partially through DAP10 to engage partially divergent downstream modules involving SYK/PI3K, PLCγ, Ras/ERK, mTORC1/2–AMPK–ULK1, β-catenin, and actin remodeling. These modules are shown schematically and do not imply equal evidentiary weight or exhaustive pathway coverage. Collectively, they shape cargo clearance, phagocytosis, metabolic fitness, inflammatory gene control, and TREM2-linked state genes, thereby influencing microglial state transition and survival. ADAM10/17-mediated shedding generates soluble TREM2 (sTREM2), and the magnitude and direction of TREM2 output are modified by disease stage, receptor dosage, ligand composition, and local context. The figure was created using Figdraw. Aβ, amyloid-β; AD, Alzheimer's disease; ADAM10/17, a disintegrin and metalloproteinase 10/17; AMPK, AMP-activated protein kinase; ApoE, apolipoprotein E; DAMPs, damage-associated molecular patterns; DAP10/12, DNAX-activating protein of 10/12 kDa; DAG, diacylglycerol; ERK, extracellular signal-regulated kinase; IP3, inositol 1,4,5-trisphosphate; mTORC1/2, mechanistic target of rapamycin complex 1/2; PI3K, phosphoinositide 3-kinase; PIP2/3, phosphatidylinositol 4,5-bisphosphate/phosphatidylinositol 3,4,5-trisphosphate; PLCγ, phospholipase C-γ; sTREM2, soluble TREM2; SYK, spleen tyrosine kinase; TREM2, triggering receptor expressed on myeloid cells 2; TYROBP, TYRO protein tyrosine kinase binding protein; ULK1, unc-51-like kinase 1; CST7, cystatin F; SPP1, secreted phosphoprotein 1. Created by Figdraw.com.

INVOKE-2 is most useful as a warning against linear expectations. Its failure shows how strongly response can depend on when, how strongly, and in what biological setting TREM2 is engaged (111). Preclinical work reinforces that point, with different TREM2 agonist antibodies producing neutral or even detrimental effects across mixed-sex Alzheimer's and multiple sclerosis models (112). Pathological background is one source of this dependence. Chronic TREM2 activation aggravates Aβ-associated tau seeding and spreading, so an Aβ-only setting cannot be assumed to share a therapeutic direction with an Aβ-plus-tau setting (113). Disease stage is another determinant. PU.1 drives β-amyloid-induced TREM2 upregulation in microglia and shows that the baseline available for therapeutic manipulation is itself remodeled as pathology accumulates (114). Local cellular environment also matters. A late-stage SEMA6D-TREM2 axis reorganizes neuron–microglia cross-talk, making apparently similar levels of receptor engagement interpretable in different ways under different niche conditions (115). Dose cannot be reduced to administered amount alone. The potency and *in vivo* pharmacodynamic behavior of a biparatopic TREM2 agonistic antibody depend on receptor-clustering geometry and molecular format (116). Modality belongs in the same framework: first-in-class direct small-molecule agonists activate TREM2 through a distinct pharmacologic route, so antibody-derived results should not be generalized into a single rule for all TREM2 agonism (117) (Figure 3). More broadly, emerging work on adaptive immune traffic across the blood–brain barrier indicates that CNS immune modulation may depend on interactions extending beyond microglia alone, reinforcing the need to interpret TREM2-directed strategies within a wider neuroimmune context rather than as a single-receptor solution (118).

## 6 Human readouts of substrate-linked microglial metabolic fitness

### 6.1 Principles for interpreting biomarkers

Biomarker interpretation should begin with cell specificity. Analyte class, disease stage, cohort structure, and assay context all shape what an individual signal can reasonably mean in Alzheimer's disease (16). Matrix, assay, and stage matter in the same way. Even clinically informative human associations cannot be converted into causal or one-to-one maps of brain-resident microglial states, and current guidance on inflammatory fluid markers treats those limits as part of responsible biomarker use (19). The same caution applies to widely used glial readouts. In the Greater-Bay-Area Healthy Aging Brain Study (GHABS) cohort, plasma glial fibrillary acidic protein (GFAP) is interpreted alongside Aβ positron emission tomography (PET), tau PET, hippocampal atrophy, and AD-signature cortical thinning, showing that a fluid glial signal can be informative while remaining strongly cell-biased and compartment-limited (119). Stage sensitivity is better framed as the changing behavior of process-linked proxies across the Alzheimer continuum. CSF proteome profiling identifies sequential shifts in protein clusters tied to different brain-cell and pathological processes without cell-resolved proof (120). Substrate linkage requires the same restraint. A blood fatty-acid lipidome score can associate with Alzheimer risk, cognition, hippocampal volume, and CSF markers while still remaining a peripheral metabolic readout (121).

### 6.2 Fluid biomarkers

Fluid biomarkers become most informative when anchored in staged myeloid biology. CSF microglia-linked protein profiles shift across clinical stages and place even familiar markers such as sTREM2 inside a broader, time-dependent response landscape (122). Matrix shapes meaning as well. Higher plasma sTREM2 is associated with reduced cerebral tau accumulation, and that relationship cannot be transferred uncritically across blood and CSF (123). Longitudinal sampling therefore matters. The longitudinal trajectory of CSF sTREM2 in the Alzheimer's disease Neuroimaging Initiative cohort shows that interpretation depends not only on level but also on timing along the disease course (124). sTREM2 most plausibly serves as an early-response proxy, with associations to amyloid-related p-tau increases and glucose hypermetabolism pointing to a particular phase of evolving pathology (125). Lysosomal and myeloid coupling follows the same logic. Progranulin and sTREM2 remain interrelated across the AD continuum and in non-AD contexts, supporting a linked myeloid-lysosomal readout axis (126). Not all useful fluid markers are microglial, however. GFAP and YKL-40 (YKL-40) fit more naturally as astrocyte-biased progression readouts, which makes them useful for staging glial reactivity while limiting claims of microglial specificity (127). Glycoprotein non-metastatic melanoma protein B (GPNMB) sits under a similar boundary, functioning more convincingly as a candidate signal of myeloid or lysosomal stress than as a stand-alone discriminator of Alzheimer's disease (128). These limits help explain why panel logic often outperforms single-analyte logic. Multiplex CSF proteomics already supports diagnosis and prediction by integrating partially overlapping signals that would be overread if each marker were treated as self-sufficient (129). The same panel logic appears outside a single dataset. An independent CSF protein panel also supports diagnostic and predictive assessment (130). Even so, stage sensitivity still needs a disease-context boundary. CSF proteomics in autosomal dominant Alzheimer's disease maps one genetically defined continuum and cannot simply be assumed to reproduce the full temporal grammar of sporadic Alzheimer's disease (131). Fluid lipidomics adds substrate-relevant information without removing that boundary. Targeted plasma lipidomic fingerprinting remains a peripheral compartment readout (132). Fluid metabolomics should be read with the same discipline, and oxidative-stress and inflammatory metabolites in MCI still function as biochemical process correlates at the fluid level and do not provide cell-resolved proof of microglial metabolic state in brain tissue (133).

### 6.3 Imaging biomarkers

Translocator protein (TSPO) PET remains the most mature inflammation-linked imaging tool, yet its interpretability still rests on modality-level validation instead of Alzheimer-specific cellular proof (134). Within the Alzheimer continuum, 11C-ER176 TSPO PET shows stage-dependent neuroinflammatory signal alongside amyloid and tau burden, giving it genuine *in vivo* staging value without making it a microglia-exclusive or state-resolved marker (135). TREM2 PET moves imaging closer to a pathway-oriented myeloid signal. Copper-64-labeled TREM2 antibodies allow non-invasive tracking of TREM2 expression in preclinical Alzheimer models (136). Even so, current TREM2 PET evidence remains closer to model-based feasibility than to validated human Alzheimer imaging. Related work has mainly visualized TREM2 in Aβ-induced neuroinflammation systems (137). Magnetic resonance imaging (MRI)-linked readouts work better as microstructural proxies than as cell-resolved inflammation maps. Free-water diffusion can be integrated with plasma biomarkers, diffusion-based microstructural indices, macrostructural MRI measures, and cognition to capture inflammation-sensitive extracellular change (138). That caution remains necessary even at higher field strength. Plasma p-tau181 and GFAP only reflect 7T MRI-derived structural, functional, and spectroscopic changes, so MRI-based inflammatory inference still depends on multimodal anchoring (139).

### 6.4 Human tissue multi-omics and spatial profiling

Human brain multi-omics and spatial profiling provide the closest *ex vivo* anchor to brain-resident microglial states, with spatial proteomics resolving Alzheimer-specific human microglial programs *in situ* (140). Atlas-level integration adds interpretive stability across cohorts. HuMicA harmonizes disease-associated human microglial subsets across neurodegenerative datasets and shows expansion of GPNMB-high populations in Alzheimer's disease (141). Spatial mapping adds more than simple localization. A spatially resolved Alzheimer hippocampal atlas reveals subregion-specific molecular reorganization and ordered glial enrichment around plaque-bearing tissue (142). Even within that frame, plaque-adjacent microglial ecology does not collapse into a single label. Combined single-cell and spatial transcriptomics place activated microglia nearer amyloid plaques and more homeostatic populations farther away within the same diseased cortex (143). These approaches still function best as *ex vivo* reference maps. Their interpretive strength depends on tissue-based assignment, spatial resolution, and processing constraints (144).

### 6.5 Biomarkers and substrate–state axes

Biomarkers map back onto substrate–state axes most cleanly when the starting point is a biological-process panel. Inflammatory markers change meaning with sampling matrix, disease context, and time (19). GFAP- and YKL-40-dominant readouts therefore fit more naturally on a microglia-conditioned Aβ-astrocyte relay axis. Multimodal human data link Aβ-associated astrocyte reactivity most tightly to settings in which microglial activation is also present (145). Metabolic biomarkers require the same level of stratification. Sex- and APOE-specific network analysis shows that distinct patient subgroups occupy distinct metabolic signatures and driver structures (146). Plasma lipid panels fit more comfortably on a peripheral lipid-handling substrate axis than on direct brain-resident microglial lipid burden. Lipid-metabolism-based signatures can classify Alzheimer's disease accurately while remaining compartmentally peripheral (147). For substrate-linked biomarkers, longitudinal trajectories often map the axis more faithfully than a single concentration. Plasma lipidome changes over time stratify late-onset Alzheimer risk while co-varying with core biomarker and neurodegeneration-related measures (148) (Table 2).

**Table 2:** Human readouts of substrate-linked microglial metabolic fitness.

Readout	Matrix/modality	Linked axis	Most informative stage	Main use	Key limitation	References
Spatial microglial proteomics	Postmortem multiplexed ion beam imaging	Direct human state continuum	Established AD tissue	Spatial state validation	*Ex vivo* only	(140)
Microglia-enriched CSF protein profile	CSF proteomics	Stage-shifted microglial programs	Preclinical to dementia	Stage mapping	Indirect CNS proxy	(122)
Plasma sTREM2	Plasma immunoassay	Peripheral TREM2 signal linked to tau burden	Aβ-positive and tau-positive/tau-vulnerable stages	Accessible blood readout	Not microglia-specific	(123)
Longitudinal CSF sTREM2	CSF immunoassay	TREM2-linked trajectory	Preclinical to early symptomatic AD	Longitudinal tracking	Age-sensitive, non-spatial	(124)
CSF sTREM2 with FDG-PET/p-tau coupling	CSF + PET	Early Aβ-linked microglial response	Early Aβ-positive stages	Stage-sensitive mechanistic linkage	Sample/stage complexity	(125)
CSF PGRN–sTREM2 coupling	CSF immunoassay	Lysosome–microglia axis	tau/neurodegeneration-positive/inflammatory continuum	Cohort-level coupling	Not cell-specific	(126)
GFAP/YKL-40 staging	Plasma + CSF	Astrocyte reactivity downstream of Aβ/injury	GFAP early; YKL-40 later	Stage-sensitive glial readout	Astrocyte-biased	(127)
Microglia–astrocyte coupling readout	Multimodal human study	Microglia-conditioned astrocyte reactivity	Aβ-positive to tau-vulnerable stages	Cell-interaction interpretation	Not a standalone biomarker	(145)
CSF GPNMB	CSF immunoassay	Lysosomal/myeloid stress	Likely later inflammatory stages	Biologically plausible stress readout	Weak diagnostic performance	(128)
TSPO PET with postmortem validation	PET + pathology	Activated glial burden	Symptomatic tauopathy/AD-relevant contexts	*In vivo* regional mapping	Not cell-specific	(134)
11C-ER176 TSPO PET	PET	Neuroinflammatory burden	Preclinical A+ to symptomatic AD	AD-stage mapping	Burden > phenotype	(135)
TREM2 PET with copper-64 antibody	PET	TREM2-positive myeloid response	Preclinical/model stage	Target-proximal imaging	No mature human AD data	(136)
TREM2 PET in amyloid-driven neuroinflammation	PET	TREM2-positive response	Preclinical/model stage	Imaging feasibility	Preclinical only	(137)
Human microglia atlas	Postmortem atlas	Human disease-associated/GPNMB-high microglial states	Cross-disease anchoring	State interpretation scaffold	*Ex vivo* only	(141)
Multiplex CSF proteomic panel	CSF proteomics	Composite microglia-linked and broader AD biology	Preclinical to dementia	Diagnosis/prediction	Limited cell specificity	(129)
Longitudinal CSF proteomic staging	CSF proteomics	Natural history of AD biology	Preclinical to symptomatic AD	Temporal staging	Not microglia-specific	(131)
CSF diagnostic protein panel	CSF proteomics	Broad AD molecular signature	Symptomatic and prodromal AD	Diagnostic/predictive panel	Limited mechanistic specificity	(130)
Plasma metabolic network signature	Plasma metabolomics	Sex/APOE-modified metabolic axis	At-risk/MCI/AD stratification	Subgrouping	Peripheral and indirect	(146)
Plasma lipid-metabolism biomarker panel	Plasma metabolomics	Lipid-handling signature	At-risk/MCI/AD stratification	Classification/stratification	Peripheral and indirect	(147)

This table summarizes human fluid, imaging, and tissue readouts relevant to substrate-linked microglial metabolic fitness across the Alzheimer's disease continuum. These measures should be interpreted as stage- and context-sensitive proxies rather than direct one-to-one representations of brain-resident microglial states. “Most informative stage” denotes the disease window in which a readout is likely to be most informative on current evidence, whereas “Key limitation” identifies the principal boundary on mechanistic or translational interpretation. References are representative rather than exhaustive, with one key study listed per row. Representative intervention relevance is summarized in Table 3. AD, Alzheimer's disease; Aβ, amyloid-β; APOE, apolipoprotein E; CNS, central nervous system; CSF, cerebrospinal fluid; FDG-PET, fluorodeoxyglucose positron emission tomography; GFAP, glial fibrillary acidic protein; GPNMB, glycoprotein non-metastatic melanoma protein B; MCI, mild cognitive impairment; PET, positron emission tomography; PGRN, progranulin; p-tau, phosphorylated tau; sTREM2, soluble TREM2; TSPO, translocator protein.

## 7 From nutritional substrate biology to translation

### 7.1 Interventions and disease windows

Nutritional substrate interventions fit most naturally in at-risk, MCI, or early symptomatic populations, where host-level metabolic rescue can be measured before late-stage neurodegeneration obscures interpretation (83). Ketogenic medium-chain triglycerides also increase brain ketone uptake and total brain energy metabolism in mild-to-moderate Alzheimer's disease, extending that substrate-response logic into symptomatic disease (82). Receptor-directed microglial modulation currently fits biomarker-confirmed early Alzheimer's disease better than undifferentiated late-stage disease (149). Active amyloid clearance maps to a different window, most naturally to amyloid-positive symptomatic stages in which microglial mechanisms of Aβ removal are most clearly engaged (150). Donanemab trials likewise place the clearest present-day anti-amyloid window in early symptomatic disease enriched for amyloid and tau biology (151). The same therapeutic class is now moving earlier, because TRAILBLAZER-ALZ 3 recruits biomarker-positive individuals in the preclinical stage, although that shift is still best read as a prevention-window strategy under evaluation (152). Across these intervention classes, window assignment works best as a biology-first exercise rather than a drug-first exercise (153).

### 7.2 Nutritional strategies and microglia-targeted therapies

In a combination framework, anti-amyloid treatment can provide a directly plaque-clearing microglial arm (154). Nutritional intervention sits more naturally as a substrate-context modifier, fitting a broader metabolic-context framework better than a second plaque-clearing effector model (95). At the mechanistic level, BHB reprograms human microglial metabolism, suppresses Aβ oligomer-induced inflammatory activation, and restores phagocytic competence (14). At the host level, a modified Mediterranean-ketogenic diet alters the gut microbiome and short-chain fatty acids in association with CSF Alzheimer markers in MCI (155). These findings support combination logic, but they do not yet establish that nutritional strategies improve the efficacy of microglia-targeted or anti-amyloid therapies in patients. Accordingly, nutritional plus microglia-targeted therapy is better treated as a biomarker-guided adjunctive hypothesis than as an established combination regimen (156). Representative nutritional and related microglia-targeted intervention classes, their disease-stage fit, microglial readouts, and principal caveats are summarized in Table 3.

**Table 3:** Stage-stratified nutritional and related translational interventions relevant to microglial metabolic fitness in Alzheimer's disease.

Intervention	Model/system	Disease stage	Outcome	Microglial readout	Key limitation	References
Ketogenic diet	Female APOE4 mouse model	Preclinical, genotype-defined stage	Improved memory performance	No direct microglial endpoint	Female-specific, genotype-specific mouse model; human microglial relevance remains indirect	(93)
Ketogenic medium-chain triglycerides (MCT)	Mild–moderate AD PET metabolic study	Mild–moderate AD	Brain ketone uptake and total brain energy metabolism increased	No direct microglial endpoint	Brain energetics improved, but microglial-state tracking was not performed	(82)
Ketogenic drink (kMCT)	6-month randomized controlled trial in MCI	MCI/early symptomatic stage	Improved cognitive outcomes	No direct microglial endpoint	Clinical benefit was not linked to direct microglial biomarkers	(83)
BHB substrate exposure	Human iPSC-derived microglia challenged with Aβ oligomers	Preclinical mechanistic/early symptomatic rationale	Suppressed inflammatory activation and restored phagocytic competence	Direct human microglial mechanistic readout	Cell-model evidence; not direct *in vivo* human brain evidence	(14)
Modified Mediterranean ketogenic diet (MMKD)	Randomized crossover dietary trial in at-risk older adults with MCI or subjective memory complaints and prediabetes	At-risk/preclinical/MCI-related stage	Reversed an AD-like peripheral lipid signature	No direct microglial endpoint	Peripheral lipidomic readout; brain-resident microglial effects remain inferred	(7)
Omega-3/DHA-containing intervention (MCT + DHA supplementation)	Randomized double-blind placebo-controlled trial in older adults with MCI	MCI/early symptomatic stage	Improved cognitive function	No direct microglial endpoint	Combined intervention; DHA-specific and microglial mechanisms cannot be isolated	(92)
AL002 first-in-human TREM2 agonism	Cynomolgus monkey studies plus phase 1 healthy-volunteer trial	Early translational/early AD rationale	Dose-dependent CSF sTREM2 reduction and pharmacodynamic biomarker changes	Indirect microglial pharmacodynamic readout	No clinical efficacy outcome	(103)
AL002 phase 2 TREM2 agonism	Randomized, double-blind, placebo-controlled trial in early AD	Aβ-positive MCI/mild AD dementia	Sustained target engagement and pharmacodynamic response, but primary endpoint negative	Indirect pharmacodynamic biomarker readout	No clinical benefit; ARIA-like MRI changes were frequent	(149)
BBB-enhanced TREM2 agonism	Human iPSC-derived microglia and AD mouse models	Early amyloid-positive stages	Improved brain exposure, enhanced signaling, and increased microglial metabolism and brain glucose uptake	Direct preclinical microglial metabolic and state readout	Preclinical only	(104)
TREM2 expression–response window	TREM2 reporter mice and APP transgenic microglia	Preclinical amyloid-associated stages	TREM2 level tracked with metabolic capacity, cholesterol homeostasis, and phagocytic competence; mid-level cells were most responsive to agonism	Direct metabolic, lipidomic, and phagocytic microglial readout	Clinical state-matching remains unresolved	(5)
Biparatopic TREM2 agonistic antibody	*In vitro* signaling assays and *in vivo* pharmacodynamic study	Preclinical platform-development stage	Higher potency and efficacy in signaling assays; TREM2-mediated chemokine responses *in vivo*	Direct signaling and pharmacodynamic readout	No AD efficacy outcome	(116)
Neutral or detrimental TREM2 agonism	Mixed-sex mouse models of AD pathology and demyelination	Model- and timing-dependent chronic dosing context	Limited impact on AD pathology and worsened recovery after demyelination	Pharmacodynamic plus pathology readouts	Benefit is not guaranteed and may be context-dependent	(112)
Active/passive Aβ immunization in human tissue	Human immunized AD postmortem tissue with spatial transcriptomics	Symptomatic Aβ-positive AD with established plaque burden	Distinct microglial states associated with Aβ clearance; APOE/TREM2 upregulation and complement involvement	Direct human spatial microglial readout	Postmortem evidence; not prospective monitoring evidence	(150)
Lecanemab-induced amyloid-clearing program	Human microglia xenograft mouse model	Mechanistic relevance to symptomatic Aβ-positive AD	Reduced Aβ pathology and neuritic damage; induced phagocytic, lysosomal, and metabolic programs	Direct single-cell and spatial microglial readout	Preclinical mechanism only	(154)
Microglial lipid-load reduction	APP-KI/Fit2iΔMϕ mouse model with inducible FIT2 depletion in CX3CR1+ brain macrophages	Preclinical amyloid pathology	Reduced lipid-droplet burden, enhanced Aβ phagocytosis, and reduced plaque load	Direct microglial lipid-load and phagocytosis readout	Preclinical, male HFD-fed model; genetic rather than dietary intervention	(55)

This table summarizes stage-stratified nutritional interventions and related translational comparators relevant to microglial metabolic fitness in Alzheimer's disease. Nutrition-focused entries are listed first, followed by related microglia-targeted and amyloid-clearing comparators that provide translational context for Section 7. “Microglial readout” indicates whether the study directly measured microglial or microglia-like responses. Human dietary, animal-model, postmortem, and in vitro evidence should not be interpreted as equivalent. AD, Alzheimer's disease; Aβ, amyloid-beta; BHB, β-hydroxybutyrate; CSF, cerebrospinal fluid; DHA, docosahexaenoic acid; iPSC, induced pluripotent stem cell; kMCT, ketogenic medium-chain triglyceride; MCI, mild cognitive impairment; MCT, medium-chain triglyceride; MMKD, modified Mediterranean ketogenic diet; PET, positron emission tomography; sTREM2, soluble TREM2.

### 7.3 Priorities for future trials and mechanistic studies

The first priority for future trials is to determine which neuroinflammatory biomarkers remain reproducible across cohorts and disease stages, because analyte class, cohort structure, and assay context still drive substantial heterogeneity (16). Standardization therefore has to be built into trial design from the start, and inflammatory fluid markers should not be treated as interchangeable surrogates when their interpretation depends on sampling matrix, assay platform, and disease stage (19). A second priority is to determine whether peripheral markers can do more than enrich cohorts—whether they can actually stratify brain-resident microglial responses relevant to intervention selection; plasma GFAP has already been proposed as one enrichment tool for preclinical Alzheimer's disease (157). A third priority is to move amyloid-related imaging abnormalities from an after-the-fact safety issue to a protocol-level design variable across anti-amyloid therapies (158). In practice, APOE ε4 status and baseline imaging findings already shape donanemab-associated ARIA risk (159). A fourth priority is to test whether a single baseline biomarker can help stratify both likely benefit and likely harm, as higher baseline CSF p-tau181 during lecanemab treatment has been associated with greater cognitive decline and higher ARIA occurrence in clinical-practice data (160). More fundamentally, future studies still need to establish whether dietary substrates truly modify microglial metabolic programs in humans, whether APOE genotype changes nutritional responsiveness, and whether ketone- or lipid-based interventions should be tested as adjuncts rather than stand-alone disease-modifying therapies.

## 8 Discussion

Alzheimer's disease is best understood here as a staged redistribution of microglial states, in which functional fit is progressively lost as pathology advances within the same lineage (2). Microglial metabolic fitness is useful because it links substrate handling to disease-relevant function rather than to activation labels alone: energy production, glucose uptake, lipid-cargo handling, lysosomal competence, phagocytosis, and TREM2-linked state transition become parts of the same biological problem (5). Human readouts should therefore be treated as time- and compartment-sensitive proxies, not as fixed signatures of a single microglial state. CSF microglia-associated protein profiles shift across clinical stages, underscoring that biomarker meaning changes with disease timing (122). The translational premise is no longer purely theoretical; lecanemab-associated plaque reduction has been linked to an amyloid-clearing microglial program, indicating that disease-relevant microglial states can be shifted therapeutically (154). The harder task is to map substrate-linked microglial states onto readable biomarkers and stage-appropriate interventions with enough precision to match the right biology to the right patient and disease window (161).

The central translational gaps are narrower, but more demanding, than whether nutritional interventions are biologically active. Ketogenic medium-chain triglycerides increase brain ketone uptake and total brain energy metabolism in mild-to-moderate Alzheimer's disease (82), and a 6-month ketogenic drink improves several cognitive outcomes in MCI (83). These studies establish host- and brain-level substrate responsiveness, but they do not show that dietary substrates reprogram brain-resident microglia in living humans. Direct substrate-to-microglia evidence still comes mainly from cell systems, where BHB suppresses Aβ oligomer-induced inflammatory activation and restores phagocytic competence in human microglia (14). Whether dietary substrates reach or reshape brain-resident microglial metabolic programs in patients therefore remains unresolved. Peripheral biomarkers face the same boundary. Plasma lipidome trajectories stratify late-onset Alzheimer risk over time, but whether such trajectories stratify brain-resident microglial responses, rather than mainly indexing systemic or disease-level risk, remains uncertain (148). Patient biology is also unlikely to follow a single rule: sex- and APOE-specific metabolic networks suggest that APOE genotype may condition nutritional responsiveness (146). These gaps argue for testing ketone- and lipid-based interventions, at least initially, as adjunctive or context-conditioning strategies alongside disease-modifying therapies rather than as stand-alone disease-modifying therapies.

Microglial metabolic failure is also unlikely to remain confined to microglia. At the neurovascular interface, endothelial dysfunction, blood–brain barrier vulnerability, oxidative stress, and PI3K/AKT/mTOR-linked vascular signaling form a broader pathophysiological nexus that can amplify neurodegeneration (162). Systemic endocrine-metabolic frameworks point in the same direction: AMPK activation, mTOR restraint, autophagy restoration, and suppression of senescence-associated inflammatory signaling may shape how strongly aging brains tolerate chronic glial stress (163). These links do not prove that dietary substrates directly remodel human microglial states. They do, however, define the host environments in which substrate-linked microglial dysfunction may be amplified, buffered, or made measurable. Microglial metabolic fitness should therefore be interpreted within neurovascular and systemic metabolic loops, not as an isolated cellular property.

Future work should move from broad nutritional exposure to biomarker-defined, stage-aware intervention logic. Human studies need high-resolution, cell-specific multi-omic maps that connect substrate-linked microglial states with fluid, imaging, and tissue readouts across disease stage, APOE genotype, sex, and vascular-metabolic burden. Nutritional strategies should be tested as modifiers of systemic substrate tone, inflammatory set point, and treatment context, while natural or synthetic multi-target agents should be evaluated for their ability to restore lysosome–mitochondria coupling, lipid handling, and inflammatory-resolution programs under disease-relevant stress. The next frontier is not a single diet, substrate, or microglial target, but an integrated framework in which dietary interventions, secondary metabolites, endocrine homeostasis, and neurovascular tracking are used to align substrate logic with patient biology and therapeutic window.
